# Various interventions for cancer-related fatigue in patients with breast cancer: a systematic review and network meta-analysis

**DOI:** 10.3389/fonc.2024.1341927

**Published:** 2024-02-09

**Authors:** Ying Li, Lei Gao, Yaqing Chao, Tianhao Lan, Jie Zhang, Ruoqi Li, Zerui Zhang, Shuming Li, Jing Lian, Zhaofeng Wang, Xiaoan Chen

**Affiliations:** ^1^College of Sports Science, Jishou University, Jishou, Hunan, China; ^2^School of Nursing, Dalian University, Dalian, Liaoning, China; ^3^Ophthalmology Department, Xuzhou First People’s Hospital, Xuzhou, Jiangsu, China; ^4^School of Stomatology, Dalian University, Dalian, Liaoning, China; ^5^The Third Hospital of Shanxi Medical University, Taiyuan, Shanxi, China; ^6^Medical School, Weifang University of Science and Technology, Weifang, Shandong, China; ^7^Department of Pathology, Cancer Hospital Affiliated to Shanxi Province Cancer Hospital, Taiyuan, Shanxi, China; ^8^College of Physical Education, Beibu Gulf University, Qinzhou, Guangxi, China

**Keywords:** breast cancer, CFR, network meta-analysis, cancer, systematic review

## Abstract

**Purpose:**

To investigate the effects of various intervention approaches on cancer-related fatigue (CRF) in patients with breast cancer.

**Method:**

Computer searches were conducted on PubMed, Embase, Cochrane Library, Web of Science, China National Knowledge Infrastructure (CNKI), China Science and Technology Journal Database (VIP), and Wanfang databases from their establishment to June 2023. Selection was made using inclusion and exclusion criteria, and 77 articles were included to compare the effects of 12 interventions on patients with breast cancer.

**Results:**

Seventy-seven studies with 12 various interventions were examined. The network findings indicated that cognitive behavioral therapy (CBT) (SMD, -1.56; 95%CI, -3.08~-0.04), Chinese traditional exercises (CTE) (SMD, -0.85; 95%CI, -1.34~-0.36), aerobic exercise (AE) (SMD, -0.77; 95%CI, -1.09~-0.45), multimodal exercise (ME) (SMD, -0.75; 95%CI, -1.26~-0.25), music interventions (MI) (SMD, -0.74; 95%CI, -1.45~-0.03), and yoga (YG) (SMD, -0.44; 95%CI, -0.83 to -0.06) can reduce CRF more than the control group (CG). For relaxation exercises (RE) (MD, -6.69; 95%CI, -9.81~-3.57), MI (MD, -5.45; 95%CI, -7.98~-2.92), AE (MD, -4.34; 95%CI, -5.90~-2.78), ME (MD, -3.47; 95%CI, -4.95~-1.99), YG (MD, -2.07; 95%CI, -3.56~-0.57), and mindfulness training (MD, -1.68; 95%CI, -2.91~-0.46), PSQI improvement was superior to CG. In addition, for CTE (MD, 11.39; 95%CI, 4.11-18.66), YG (MD, 11.28; 95%CI, 1.63-20.93), and AE (MD, 9.34; 95%CI, 0.26~18.42), Functional Assessment of Cancer Therapy-Breast improvement was superior to CG.

**Conclusion:**

Cognitive behavioral therapy (CBT) is the most effective measure for alleviating CRF in patients with breast cancer and Relaxation exercises (RE) is the most effective measure for improving sleep quality. In addition, Chinese traditional exercises (CTE) is the best measure for enhancing quality of life. Additional randomized controlled trials (RCTs) are expected to further investigate the efficacy and mechanisms of these interventions.

**Systematic review registration:**

https://www.crd.york.ac.uk/prospero/, identifier CRD42023471574.

## Introduction

Breast cancer is the most common cancer in women. The American Cancer Society reports a yearly increase of 0.5% in the incidence of breast cancer in women. The projection for 2022 estimates approximately 287,850 new cases of breast cancer in women in the United States, accounting for 31% of all new cancer diagnoses in women (1). In recent years, the survival rate of patients has improved due to the emergence of neoadjuvant therapy. However, survivors face a series of physical and mental problems, such as premature menopause, body image disorder, fatigue, and depression (2–4). Patients with breast cancer commonly experience cancer-related fatigue (CRF) as one of the most common symptoms (5). Before undergoing anticancer treatment, women with breast cancer may have already experienced fatigue. The occurrence of CRF is closely connected to the factors inherent to the primary tumor, which may be associated with the abnormal expression of certain substances released by the cancer cells in the patient, such as IL1, IL6, and TNF-a interferon. The severity of fatigue is proportional to the amount of interleukin released by tumor cells into the blood (6). When starting treatment, between 60% and 90% of women with breast cancer may experience fatigue (7). Severe fatigue is experienced by approximately a quarter of breast cancer survivors (8). An increase in the burden on patients’ families and caregivers can be caused by CRF. In addition, the time it takes for patients to return to work early after cancer treatment may be prolonged by CRF (9–13).

In recent years, there has been a growing interest in investigating CRF for breast cancer. The effects of aerobic exercise (AE), resistance exercise, relaxation training, yoga (YG), music, and other intervention methods on CRF in patients with breast cancer have been investigated in previous studies. Traditional meta-analyses have also demonstrated the effectiveness of YG and resistance training (RT) in reducing CRF in patients with breast cancer (14, 15). Olsson et al. offered a comprehensive overview of the effects of rehabilitation interventions and discovered that CRF was positively affected by exercise and YG (16). Health-related quality of life in breast cancer survivors can be significantly impaired by the occurrence of CRF. Practical exercise training can enhance mitochondrial function and plasticity in patients, thereby improving the occurrence of CRF (17–19). However, evidence-based recommendations regarding the most effective type of intervention for improving CRF in patients with breast cancer are still lacking. Therefore, it is crucial to identify a suitable intervention for reducing CRF among complex interventions in patients with breast cancer.

Network meta-analysis (NMA), also called meta-analysis of mixed treatment comparisons or multiple treatment comparisons (20), offers a method to compare the size of the impact of various intervention types on CRF in patients with breast cancer by estimating direct and indirect comparisons. Although two previously published NMA studies were identified (21, 22), the study only reported on the effects of various exercise interventions, and no further studies were conducted on other intervention types. Consequently, this study aims to conduct an NMA on relevant randomized controlled trials (RCTs) to compare the effects of various interventions on CRF in patients with breast cancer. The findings of this study are crucial for developing clinical practice guidelines recommending the best intervention to improve the outcome of CRF in patients with breast cancer.

## Methods

This NMA was designed based on the guidelines for Preferred Reporting Items of Systematic Review and Network Meta-Analysis (23), which are registered in the PROSPERO database (CRD42023471574).

### Search strategies

Searches for RCT-related studies on CRF in breast cancer, published up to July 2023, were conducted using databases such as PubMed, Web of Science, Embase, Cochrane Library, China National Knowledge Infrastructure, and Wanfang. The search involved a combination of subject and free words. The search strategy can be found in Additional Document 1 (**Appendix 1**).

### Study selection

In this study, YL and LG were selected as independent reviewers to screen the titles and abstracts of the retrieved literature using search strategies to identify literature that met the inclusion criteria. In case of disagreement, checks and discussions were performed by XA C to reach a consensus. Duplicate data were de-duplicated using EndNote software (24). A full-text assessment of potentially eligible studies was conducted based on the inclusion and exclusion criteria. Any differences between the reviewers were resolved through discussion, and EndNote software was used to manage this phase.

### Inclusion criteria

Studies that met the following criteria were included (1): Study type: RCT (2). Studies that included adult patients (18 years or older) diagnosed with breast cancer that were not limited to cancer stage and current treatment options for breast cancer (3); Interventions: AE, RT, Chinese traditional exercises (CTE), other exercise (OE), multimodal exercise (ME), YG, stretching exercise (STE), music interventions (MI), cognitive behavioral therapy (CBT), mindfulness training (MT), and relaxation exercises (RE); and (4) Outcomes: at least one outcome measure. The primary outcome measure was CRF assessed using the Functional Assessment of Cancer Therapy (FACT)-Fatigue Scale, European Organization for Research and Treatment of Cancer Quality of Life Questionnaire (EORTC QLQC30), Piper Fatigue Scale (PFS), Schwartz Cancer Fatigue Scale (SCFS), and Multidimensional Fatigue Inventory (25). The secondary outcomes were sleep quality versus quality of life as measured using the Pittsburgh Sleep Quality Index (PSQI) and Functional Assessment of Cancer Therapy-Breast (FACT-B). Each intervention is defined in Additional Document 1 (**Appendix 2**). Each outcome measure is defined in Additional Document 1 (**Appendix 3**).

### Exclusion criteria

(1) Patients with severe complications (2). Studies with outcomes that did not align with the design of this study (3). Studies with data that could not be integrated, such as incorrect or incomplete information.

### Data extraction

The reviewers independently extracted the following data: first author, publication year, country, sample size, body mass index (BMI), age, weight, height, weight, intervention, tumor stage, intervention time, intervention frequency, and outcome indicators. Data are presented as mean ± standard deviation.

### Risk of bias assessment

Two reviewers (LG and ZR Z) independently assessed the risk of bias, and a third reviewer adjudicated using Cochrane collaboration tools, such as sequence generation, assignment hiding, blinding, incomplete outcome data, non-selective outcome reporting, and other sources of bias (26). Each criterion was judged to have a low, unclear, or high risk of bias (27).

### Data analysis

The “Netmeta” package (28) in R-4.2.1 software (29) was used for NMA. Network plots were generated using the STATA 15.1 “network plot” feature to describe and present various forms of motion. Nodes were used to represent various interventions, and edges were used to depict favorable intervention comparisons. Inconsistencies between direct and indirect comparisons were evaluated using the node segmentation method (30). Combined estimates and 95% confidence intervals (95% CI) were computed using random effects network element analysis. In studies where the same unit of measurement was of interest, the mean difference (MD) was considered a treatment effect when analyzing the results or evaluating the standardized mean difference (SMD). Different exercise treatments were compared using a pairwise randomized effects meta-analysis. The heterogeneity of all pair-to-pair comparisons was evaluated using the *I^2^* statistic, and publication bias was evaluated using the p-value of Egger’s test. Publication bias and secondary study effects, analyzed by the results of more than a dozen reported studies, were identified using funnel plots.

## Results

### Literature selection

**Figure 1:**
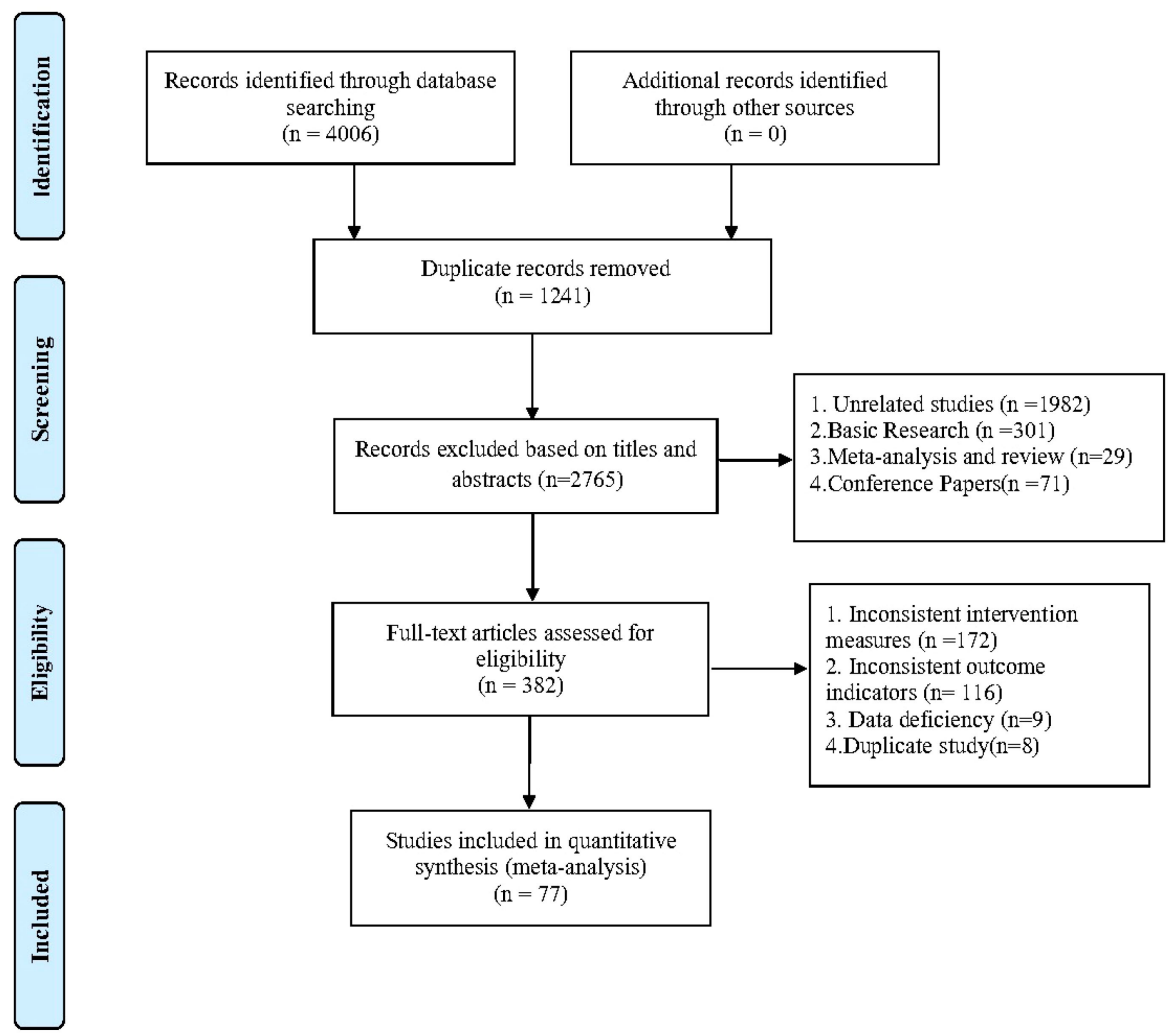
Flow of trials through the review.

After removing duplicates, 4006 records were retrieved, and 3624 papers were discarded. The full text of the remaining 382 records was analyzed, and 305 cases did not satisfy the inclusion criteria: inconsistent intervention measures (172), inconsistent outcome indicators (31), data deficiency (9), and duplicate study (8). In the end, 77 (32–108) studies were included. **Figure 1** shows the research flowchart. Articles from the full-text evaluation and the reasons for their exclusion are provided in Additional Document 1 (**Appendix 8**).

### Study and participant characteristics

Studies comparing the effects of 12 various interventions on patients with breast cancer, published between 2001 and 2022, were included. The intervention durations ranged from 1 week to 12 months, and a total of 5,254 patients were reported in the included studies. Among these studies, 62 reported CFR, 23 reported PSQI, and 17 reported FACT-B. The participants had an average age of 18-73 years, an average BMI of 21.06 ± 2.26-72.3 ± 13.1, an average height of 145.64 ± 24.07-170.2 ± 5.4 cm, and an average weight of 54.74± 6.66-74.3 ± 17.0 Kg.

**Figure 2:**
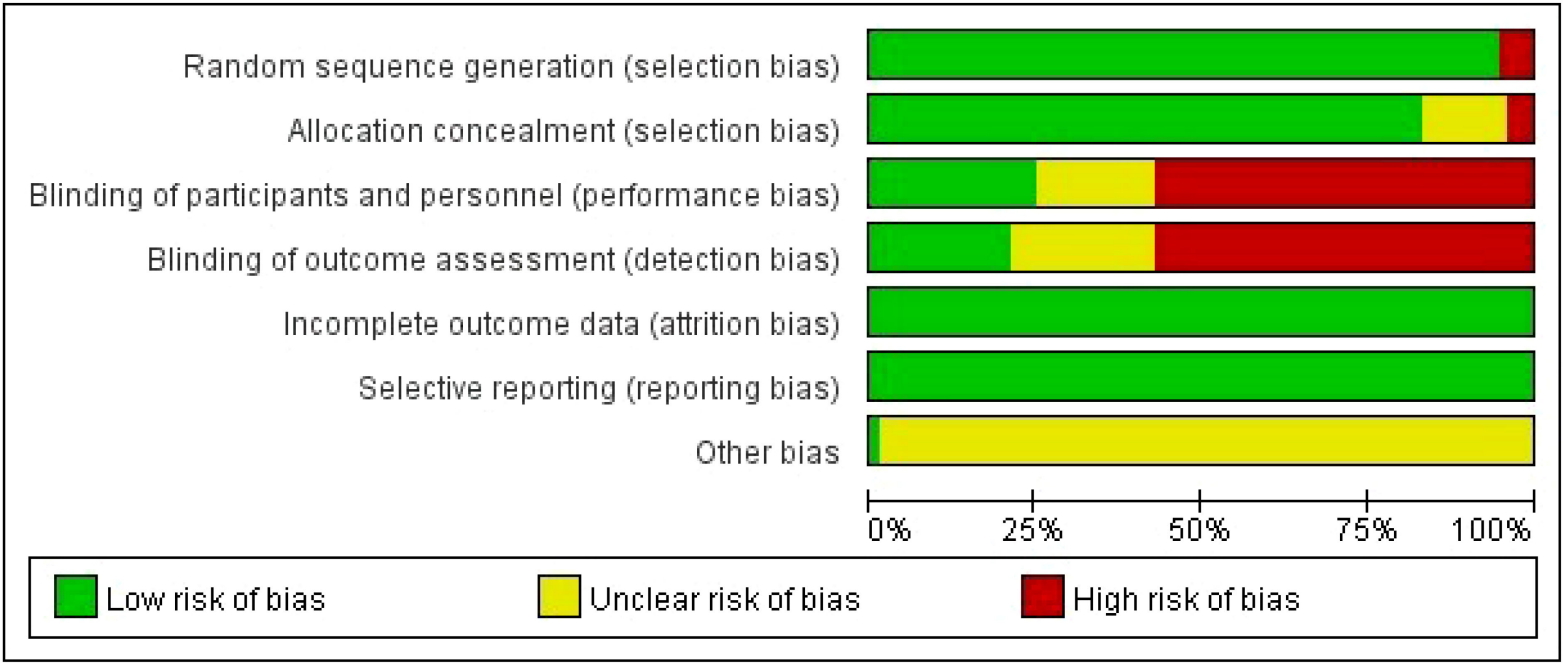
Percentage of studies examining the efficacy of interventions in patients with breast cancer with low, unclear, and high risk of bias for each feature of the Cochrane Risk of Bias Tool.

**Table 1** shows the characteristics of the studies and participants. The risk of bias assessment for each study is presented in **Additional Document 1** (**Appendix 4**), and **Figure 2** presents the aggregated data.

**Table 1:** General characteristics of all included studies.

Name	Years	Country	Group	Age	BMI	Height(cm)	Weight(kg)	Tumor staging	Sample size	Intervention time	Intervention frequency	Outcomes
Gokal (32)	2016	UK	AE/CG	52.08 ± 11.7/ 52.36 ± 8.9	27.20 ± 4.82/ 28.25± 5.83	NA	NA	I-III	25/25	12weeks	5 times a week, >20min/times	FACT-F
Pinto (33)	2005	USA	AE/CG	53.14 ± 9.70	27.01 ± 4.65/ 28.26± 5.33	NA	NA	0-II	43/43	12weeks	> 30 minutes, 5 days a week	Linear analog scale for fatigue)
Mock (34)	2005	USA	AE/CG	51.3± 8.9/ 51.6 ± 9.7	25.5± 4.0 25.8± 5.1	NA	NA	0–III	54/54	6weeks	> 60 minutes per week	PFS
Husebø (35)	2014	Norway	AE/CG	50.8 ± 9.7/ 53.6 ± 8.8	NA/NA	NA/NA	69.0 ± 11.6/ 72.0 ± 15.7	I–III	33/34	15 weeks	30 minutes/day	SCFS-6
Mock (36)	2001	USA	AE/CG	48.64 ± 10.69/ 27.95 ± 5.94	23.86 ± 3.94/ 27.95 ± 5.94	145.64 ± 24.07/ 168.59 ± 35.10	65.54 ± 2.47/ 65.18 ± 2.68	I-IIIa	28/22	NA	> 90 minutes per week	PFS
Wang (37)	2011	USA	AE/CG	48.40 ± 10.15/ 52.3± 8.84	NA/NA	156.86± 5.04/ 156.74± 4.45	55.68 ± 6.91/ 54.74± 6.66	I-II	30/32	6weeks	3-5 times a week	FACT-F/PSQI
Schmidt (38)	2015	Germany	RT/RE	52.2 ± 9.9/ 53.3 ± 10.2	25.7 ± 4.6/ 26.3 ± 4.9	NA/NA	NA/NA	0–III	49/46	12weeks	Twice a week	EORTC QLQ30-Fatigue
Steindorf (39)	2014	Germany	RT/RE	55.2 ± 9.5/ 56.4 ± 8.7	26.9 ± 5.4/ 27.6 ± 4.8	NA/NA	NA/NA	0–III	80/80	12weeks	2 times/week	EORTC QLQ30
Han (40)	2019	China	CTE/CG	46.39 ± 5.79/45.52 ± 6.50	NA/NA	NA/NA	NA/NA	I-III期	23/21	12weeks	2 times/day, 5 days/week	PFS-R
Yang (41)	2022	China	CTE/CG	NA/NA	NA/NA	NA/NA	NA/NA	I-III	43/43	12weeks	20 minutes/time, 2 times/day, 5 days/week	PFS-R/FACT-B
Hui (42)	2022	China	CTE/CG	69.51 ± 5.73	NA/NA	NA/NA	NA/NA	I-III	49/49	6 months	2 times/day, 20 minutes/time	CFS/PSQI/FACT-B
Xie (43)	2022	China	OE/AE	18∼50	22.05± 2.67/ 22.07± 3.08	NA/NA	NA/NA	I-IV	45/45	Four cycles of chemotherapy	3 ~ 4 times/week, 30 ~ 40 min/time	CFS
Xu (44)	2012	China	AE/CG	47.3±12.8	NA	NA	NA	NA	39/39	8weeks	20- 30 minutes/time	RPFS
Hao (45)	2013	China	AE/CG	46 ± 11.13/48 ± 11.32	NA	NA	63.74 ± 8.52/ 60.29 ± 8.26	I-III	28/28	15weeks	For the first 3 weeks, 3 times a week, 15min each time, then increase by 5min every 3 weeks, and reach 35min in 13-15 weeks.	RPFS
Mijwel S (46)	2018	Sweden	AE/CG	54.4± 10.3/ 52.6 ± 10.2	NA	165.3 ± 6.6/ 166.4± 7.0	67.7 ± 13.0/ 69.1± 11.0	I-III	70/60	16weeks	Twice a week	CRF
Cohen (47)	2021	USA	ME/AE/RE	59.71 ± 6.99/58.56 ± 10.41/ 53.62± 8.03	28.21± 5.39/ 26.04 ± 3.91/ 27.93± 6.35	158.2 ± 40.65/ 140.2 ± 23.07/ 162.08 ± 38.90	64.00 ± 1.93/ 63.46 ± 2.78/ 64.58 ± 2.64	I-III	13/14/13	NA	90min, three times/week.	PFS
Courneya (48)	2007	Canada	AE/RT/CG	49/49.5/49	26.7 ± 5.6/ 26.1 ± 5.5/ 27.1 ± 5.4	NA	69.4 ± 13.3/ 69.7 ± 14.4/ 72.6 ± 15.2	I-III	78/82/82	17weeks	Three times/week	FACT-F
Moadel (49)	2007	USA	YG/CG	55.11 ± 10.07/54.23 ± 9,81	NA	NA	NA	I-IV	108/56	12weeks	12sessions/week, 90minutes/session	FACIT-F
Bower (50)	2012	USA	YG/CG	54.4 ± 5.7/ 53.3 ± 4.9	NA	NA	NA	0-II	16/15	12weeks	Twice/week, 90 minutes/time	FSI/PSQI
Vadiraja (51)	2017	India	YG/CG	50.54± 8.53	NA	NA	NA	NA	33/31	3 months	NA	FSI
Vardar (52)	2015	Turkey	ME/AE	49.89 ± 4.65/47.38 ± 7.57	29.16 ± 5.74/29.27 ± 5.92	NA	NA	I-II	19/21	6weeks	30 minutes/day, 3 days/week	EORTC QOL-C30 -Fatigue
Cramer (53)	2015	Germany	YG/CG	48.3 ± 4.8/ 50.0 ± 6.7	NA/NA	169.9 ± 7.3/ 170.2 ± 5.4	69.8 ± 11.9/ 74.3 ± 17.0	I-III	19/21	12weeks	90min/week	FACIT-F/FACT -B
Vadiraja (54)	2009	India.	YG/CG	NA	NA	NA	NA	II-III	44/44	6weeks	> 3 times/week	EORTC QoL C30 Fatigue
Chaoul (55)	2018	USA	YG/SE/CG	49.5 ± 9.8/ 50.4 ± 10.3/ 49 ± 10.1	NA	NA	NA	I–III	74/68/85	12weeks	75-90min/time	BFI/PSQI
Lötzke (56)	2016	YG/CG	YG/CG	51.0 ± 11.0/ 51.4 ± 11.1	NA	NA	NA	NA	45/47	12weeks	60 minutes/week	EORTC QLQ-C30 Fatigue
Banasik (57)	2011	USA	YG/CG	63.33 ± 6.9/ 62.4 ± 7.3	NA	NA	NA	II-IV	9/9	8weeks	90 minutes/time	Fatigue Likert Scale
Strunk (58)	2018	Germany	OE/CG	54.2 ± 7.8/ 51.5 ± 8.4	NA	NA	NA	NA	30/21	24weeks	Twice/week, 90 minutes/time	QLQ-C30 Fatigue
Danhauer (59)	2009	USA	YG/CG	54.3 ± 9.6 /57.2 10.2	NA	NA	NA	Ductal carcinoma *in situ* -IV	13/14	10weeks	75 minutes/10 weeks	FACT-F/PSQI/FACT-B
Jong (60)	2018	Netherlands	YG/CG	51 ± 8/ 51 ± 7.3	NA	NA	NA	I-III	47/36	12weeks	Once a week	EORTC QLQ-C30, Fatigue
Wang (61)	2014	China	YG/CG	18~60	NA	NA	NA	NA	40/42	4 months	4 times/week, 1 time/day, 50min/time	CFS
Zeng (62)	2017	China	MI/YG/CG/ME	NA	NA	NA	NA	NA	20/24/23/22	4 months	Once/two days, 30 min/once, once/two days, 40 min/once, NA, once/two days, 40 min/once	CFS
Xiang (63)	2017	China	MI/YG/ME/CG	18~60	NA	NA	NA	NA	20/22/24/23	16weeks	Once every 2 days, 30 min/every 2 days, 40min/every 2 days, 40min/NA each time	CFS
Yang (64)	2020	China	ME/CG	49.17 ± 13.24/49.24 ± 12.09	NA	NA	NA	NA	79/83	1 month	2-5 times/week	PFS-R
Liu (65)	2018	China	AE/CG	55.2± 2.3/ 51.2± 3.2	NA	NA	NA	NA	30/30	5weeks	The first 4 weeks, 15 min/day, from the fifth week after 30 min, > 3 times/week	PFS-R
Li (66)	2019	China	AE/CG	48.0± 11.0/47.0 ± 10.0	NA	NA	NA	NA	46/46	1weeks	NA	PFS-R
Liu (67)	2015	China	AE/CG	18-62	NA	NA	NA	NA	34/35	8weeks	15 min/day for the first 4 weeks, 30 min/day for the last 4 weeks, > 3 times/week.	CRF/PSQI
Yu (68)	2020	China	AE/CG	44.01 ± 2.11/ 44.25 ± 2.24	NA	NA	NA	NA	44/44	6weeks	30min/each time, 4 times/week,	PFS-R
Chang (69)	2016	China	ME/CG	42.59 ± 6.37	NA	NA	NA	I=IV	51/49	18weeks	ME: 2-3times/day,	PSQI
Yu (70)	2021	China	YG/CG	38.8 ± 10.9/ 39.5 ± 10.3	NA	NA	NA	II-III	59/59	8weeks	Twice/day, 3 ~ 5 times/week	CFS
Yang (71)	2022	China	AE/CG	45. 56 ± 2. 37/ 45. 32 ± 2. 18	NA	NA	NA	NA	32/32	During chemotherapy	30 min/day, > 3 times/week	PFS-R/PSQI
Zhang (72)	2020	China	MI/CG	48.4 ± 8.19/ 45.48 ± 5.64	NA	NA	NA	I-IV	40/40	3 months	15 to 30 min/time	PFS-R/PSQI
Bolam (73)	2019	Sweden	RT/CG/AE	52.7 ± 10.3 /24.6 ± 4.8/ 24.8 ± 4.4	25.1 ± 4.3/ 24.6 ± 4.8/ 24.8 ± 4.4	NA	NA	I–IIIa	74/60	6weeks	60 minutes/time, twice a week	CRF
Park (74)	2020	Japan	ME/CG	53.21 ± 8.4/54.19 ± 9.27	NA	NA	NA	0-III	35/36	8weeks	20-45 min/day	BFI
Paulo (75)	2018	Brazil	MI/SE	63.2 ± 7.1/ 66.6 ± 9.6	66.9 ± 10.3/ 72.3 ± 13.1	154.1 ± 6.7/ 153.1 ± 4.5	NA	I-III	18/15	9 months	45 minutes, 3 times a week/2 times a week.	EORTC QLQ-BR23 Fatigue
Wang (76)	2022	China	CTE/CG	NA	22.15 ± 3.33/21.06 ± 2.26	NA	NA	NA	10/10	16weeks	3 times/week, 40min/each time	PFS-R
Wei (77)	2022	China	CTE/CG	40-75/ 40-75	22.86 ± 2.55/23.26 ± 2.56	NA	NA	I∼III	35/35	12weeks	5 times/week, 30min/each time	MFSI-SF/FACT-B
Schad (78)	2013	Germany	AE/CG	61.7± 9.4/ 59.3± 11.0	NA	NA	NA	I-III	30/30	6weeks	One hour per week	CFS/PSQI
Liao (79)	2022	China	CTE/CG	53.12± 7.02/54.63± 8.44	22.14± 2.67/ 23.37± 3.92	NA	NA	I–III	33/35	12weeks	2 times/week, 90 minutes/week	EORTC QLQ-C30-Fatigue/PSQI
Boing (80)	2017	Brazil	AE/CG	54.1 ± 7.6	NA	NA	NA	NA	8/11	12weeks	twice a week, 60 minutes/time	PFS-R
Naraphong (81)	2014	Thailand	ME/CG	46.36± 9.37/47.17± 6.87	NA	NA	NA	I–IIIa	11/12	10weeks	3-5 days/week	CRF
Irwin (82)	2017	USA	CTE/CBT	59.6 ± 7.9/ 60.0 ± 9.3	25.6 ± 4.5/ 26.2 ± 5.9	NA	NA	NA	38/42	3 months	120 times per week	PSQI/MFSI-SF
Chen (83)	2013	China	CTE/CG	45.3 ± 6.3/ 44.7 ± 9.7	NA	NA	NA	0-III	49/46	3 months	40 minutes, 5 times a week	BFI/PSQI
Huang (84)	2016	China	CTE/CG	NA	NA	NA	NA	NA	31/33	12 weeks	30 minutes/time	EFS
Cao (85)	2016	China	MT/CG	35.45 ± 9.21/36.12 ± 9.67	NA	NA	NA	I-III	100/ 100	8weeks	45min/days	CFS
Jiang (86)	2019	China	ME/CG	43.48 ± 9.72/42.63 ± 9.56	NA	NA	NA	I-III	58/50	4weeks	2 times/day, 20min/each time	PSQI/CFS
Fan (87)	2021	China	MT/CG	46.32 ± 5.69 /45.26 ± 5.42	NA	NA	NA	I-III	90/90	5weeks	2 ~ 3 h/time	PFS-R/PSQI
Wang (88)	2021	China	MT/CG	49. 15 ± 8. 39/51. 00 ± 9. 94	NA	NA	NA	I-III	41/43	6weeks	Once a week, 30 ~ 60min/time	CFS/PSQI
Bower (89)	2015	USA	MT/CG	46.1/47.7	NA	NA	NA	0-III	39/32	6weeks	6 times a week	FSI/PSQI
Reich (90)	2014	USA	MT/CG	58.0 ± 10.3/ 58.2 ± 9.5	NA	NA	NA	0- III	17/24	6weeks	15-45 min/day	Fatigue
Rahmani (91)	2015	Iran	MT/CG	43.25± 3.07/44.08± 3.28	NA	NA	NA	l-III	12/12	8weeks	Once/week, 2 hours/time	FSS
Lengacher (92)	2016	USA	MT/CG	56.5 ± 10.2/57.6 ± 9.2	NA	NA	NA	0-III	167/155	6weeks	6 days/week	FSI/PSQI
Daley (93)	2007	Daley	AE/CG	51.6 ± 8.8/50.6 ± 8.7	28.5 ± 4.4/27.6 ± 4.1	NA/NA	77.2 ± 12.1/73.9 ± 11.3	NA	34/38	8weeks	3 times/week	RPFS/FACT-B
Wang (94)	2017	China	CTE/CG	50.5	NA	NA	NA	I-III	45/41	3 months	20 minutes/time	CFS/PSQI
Li (95)	2019	China	YG/CG	47.5 ± 8.2/46.7 ± 9.5	NA	NA	NA	NA	45/45	2 months	60 min/time, 3 times/week	PSQI
Xiong (96)	2019	China	ME/CG	51.8 ± 4.8/ 51.5 ± 4.6	NA	NA	NA	NA	49/49	During chemotherapy	2 times/daily. 3 times/week	PSQI/FACT-B
Yuan (97)	2020	China	RE/AE	47.87± 11.94/ 45.13± 10.77	24.02± 2.11/ 24.07± 2.52	NA	NA	I-III	47/47	12weeks	RE: It was performed once every 1d, 15 min/time AE:3times/week, 15-40 minutes/time	PSQI
Zhuang (98)	2021	China	CTE/CG	35-50	NA	NA	NA	NA	70/70	12weeks	The first week 10min/time, 3 times/week, the second week extended to 10-20min/time, 4 times/week; Time in the third week 10-20min/time, 5 times/week.	PSQI
Liu (99)	2022	China	YG/CG	NA	24.71 ± 3.69/23.69 ± 3.16	NA	NA	I-II	61/62	8weeks	90 minutes/week	FACT-B
Stan (100)	2016	USA	YG/SE	61.4 ± 7.0/ 63.0± 9.3	NA	NA	NA	0-II	18/16	12weeks	> 3 times/week	FACT-B
Rogers (101)	2015	USA	AE/CG	54.9 ± 9.3/ 53.9 ± 7.7	NA	NA	NA	I-III	105/112	3 months	> 3 times/week	FACT-B
Dieli (102)	2018	USA	ME/CG	NA	BMI≥25.0	NA	NA	0-III	50/50	16weeks	> 3 times/week	FACT-B
Jin (103)	2017	China	YG/CG	55∼73	NA	NA	NA	NA	50/50	16weeks	3 times/week, 1 h/each time	FACT-B
Du (104)	2019	China	AE/CG	50.1 ± 3.84/50.8 ± 3.00	23.17 ± 3.01/23.93 ± 2.79	NA	NA	I-III	40/40	4weeks	3 times/day, 20 minutes/time	FACT-B
Li (105)	2017	China	CTE/CG	47.31 ± 9.85/45.43 ± 10.94	NA	NA	NA	0-III	31/30	3 months	Once/day, 5day/week	FACT-B
Odynets (106)	2019	Ukraine	OE/YG/CTE	59.40 ± 1.24/59.10 ± 1.37	NA	NA	NA	NA	45/30	12 months	3 times/week, 60 minutes/day	FACT-B
Fong (107)	2013	China	CG/CTE	58.3 ± 10.1/53.8 ± 4.2	NA	155.5 ± 4.3/156.7 ± 6.0	50.4 ± 7.4/55.6 ± 8.8	NA	12/16	6 months	3 times/week, 60 minutes/time	FACT-B
Loh (108)	2014	Malaya	AE/AE	18-65	NA	NA	NA	I-II	32/31	8 weeks	Twice a day/twice a week	FACT-B

Tai Chi (TC), Yoga (YG), Music interventions (MI), Aerobic exercise (AE), Relaxation training (RE), Cognitive behavioral therapy (CBT), Mindfulness training (MT), Sling exercise (SE), Qigong (QG), Baduanjin exercise (BE), Stretching exercise (STE), Resistance training (RT), Other exercise (OE), Chinese traditional exercises (CTE), CTE (TC, QG, BE), Multimodal exercise (ME), Control group (CG).

NA, Not Applicable.

### Outcomes

#### CRF

**Figure 3:**
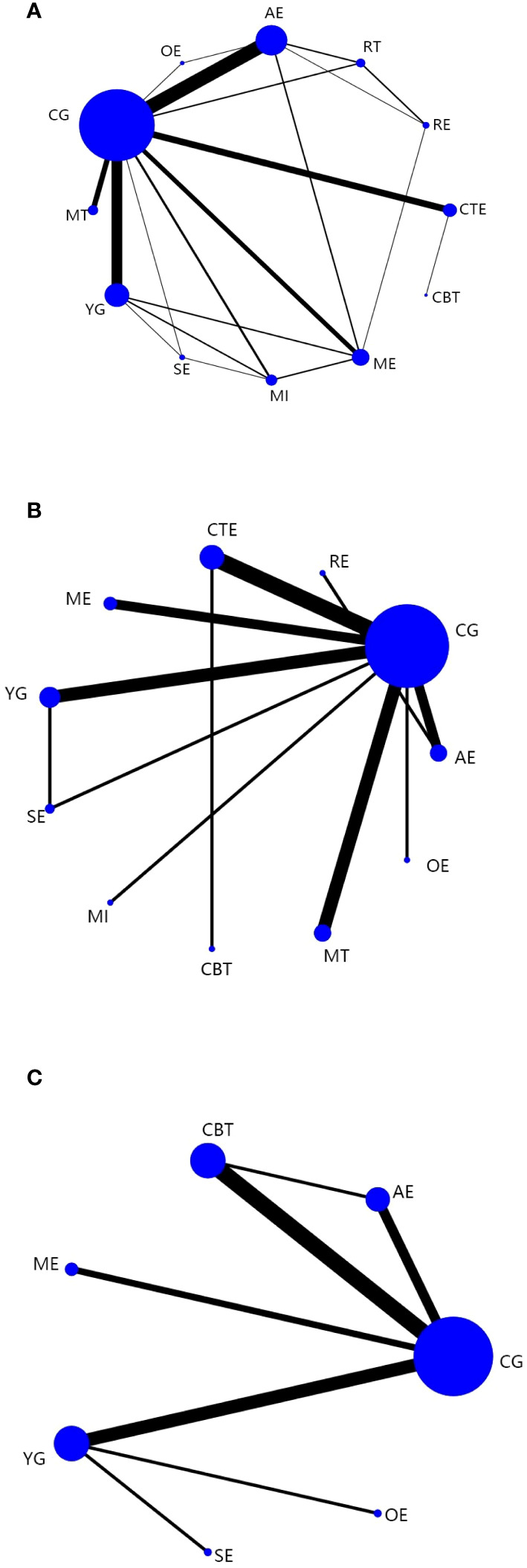
Network plots: Tai chi (TC), Yoga (YG), Music interventions (MI), Aerobic exercise (AE), Relaxation training (RE), Cognitive behavioral therapy (CBT), Mindfulness training (MT), Sling exercise (SE), Qigong (QG), Baduanjin Exercise (BE), Stretching exercise (STE), Resistance training (RT), Other exercise (OE), Chinese traditional exercises (CTE), CTE (TC, QG, BE), Multimodal exercise (ME), Control group (CG). **(A) **is network plots of CRF. **(B)** is network plots of Sleep quality. **(C)** is network plots of Quality of Life. The size of the nodes represents the number of times the exercise appears in any comparison of that treatment, and the width of the edges represents the total sample size in the comparisons it connects.

**Figure 4:**
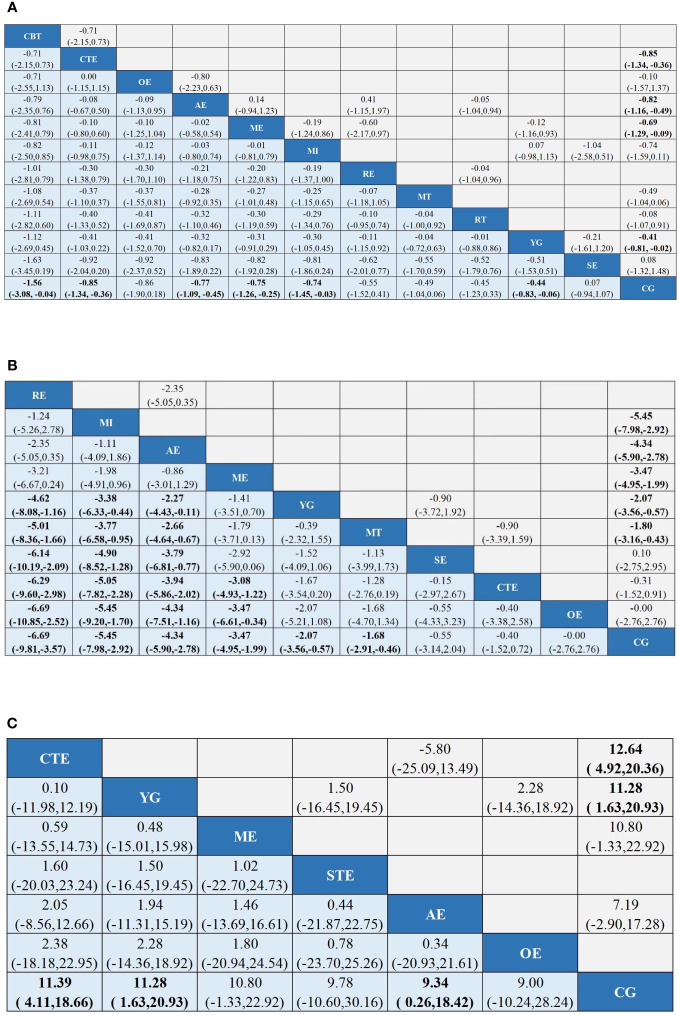
League tables of outcome analyses: Tai chi (TC), Yoga (YG), Music interventions (MI), Aerobic exercise (AE), Relaxation training (RE), Cognitive behavioral therapy (CBT), Mindfulness training (MT), Sling exercise (SE), Qigong (QG), Baduanjin Exercise (BE), Stretching exercise (STE), Resistance training (RT), Other exercise (OE), Chinese traditional exercises (CTE), CTE (TC, QG, BE), Multimodal exercise (ME), Control group (CG). **(A)** is the results of CRF's network meta-analysis. **(B) **is the results of Sleep quality's the network meta-analysis. **(C)** is the results of Quality of Life's the network meta-analysis. Data are mean differences and 95% credibility intervals for continuous data.

A total of 62 (32–94) studies, involving 5385 participants, assessed CRF. In the NMA, 12 interventions were included (**Figure 3A**): AE, CG, RT, RE, CTE, OE, ME, YG, SE, MI, CBT, and MT. Superior CFR improvement compared with CG was observed for CBT (SMD, -1.56; 95%CI, -3.08~-0.04), CTE (SMD, -0.85; 95%CI, -1.34~-0.36), AE (SMD, -0.77; 95%CI, -1.09~-0.45), ME (SMD, -0.75; 95%CI, -1.26~-0.25), MI (SMD, -0.74; 95%CI, -1.45~-0.03), and YG (SMD, -0.44; 95%CI, -0.83 to -0.06) (**Figure 4A**). Comparison of adjusted funnel plots did not provide evidence of significant publication bias, as confirmed by Egger’s test (P = 0.085) (**Additional Document 1: Appendix 5.1**). Heterogeneity, intransitivity, and inconsistencies in network meta-analyses were also evaluated (**Additional Reference 1: Appendix 6.1**). Furthermore, direct comparisons of the CRF were assessed. (**Additional Reference 1: Appendix 7.1**).

#### Sleep quality

In 23 (37, 42, 50, 55, 59, 67, 69, 71, 72, 78, 79, 82, 83, 86–89, 92, 94–98) studies, PSQI was assessed in 2334 participants. Eleven interventions were included in the NMA (**Figure 3B**): RE, MI, AE, ME, YG, MT, SE, CTE, OE, and CG. PSQI improvement was superior to YG for RE (MD, -4.62; 95%CI, -8.08~-1.16), MI (MD, -3.38; 95%CI, -6.33~-0.44), and AE (MD, -2.27; 95%CI, -4.43~-0.11). In addition, PSQI improvement was superior to MT for RE (MD, -5.01; 95%CI, -8.36~-1.66), MI (MD, -3.77; 95%CI, -6.58~-0.95), and AE (MD, -2.66; 95%CI, -4.64~-0.67). RE (MD, -6.14; 95%CI, -10.19~-2.09), MI (MD, -4.90; 95%CI, -8.52~-1.28), and AE (MD, -3.79; 95%CI, -6.81~-0.77) demonstrated superior PSQI improvement compared with SE. Furthermore, PSQI improvement was superior to CTE for RE (MD, -6.29; 95%CI, -9.60~-2.98), MI (MD, -5.05; 95%CI, -7.82~-2.28), AE (MD, -3.94; 95%CI, -5.86~-2.02), and ME (MD, -3.08; 95%CI, -4.93~-1.22). RE (MD, -6.69; 95%CI, -10.85~-2.52), MI (MD, -5.45; 95%CI, -9.20~-1.70), AE (MD, -4.34; 95%CI, -7.51~-1.16), and ME (MD, -3.47; 95%CI, -6.61~-0.34) demonstrated superior PSQI improvement compared to OE. Additionally, RE (MD, -6.69; 95%CI, -9.81~-3.57), MI (MD, -5.45; 95%CI, -7.98~-2.92), AE (MD, -4.34; 95%CI, -5.90~-2.78), ME (MD, -3.47; 95%CI, -4.95~-1.99), YG (MD, -2.07; 95%CI, -3.56~-0.57), and MT (MD, -1.68; 95%CI, -2.91~-0.46) demonstrated superior PSQI improvement compared to CG (**Figure 4**). Comparison of the adjusted funnel plot did not provide evidence of significant publication bias, as confirmed by Egger’s test (*P* = 0.744) (**Additional document 1: Appendix 5.2**). Heterogeneity, inaccessibility, and inconsistencies in the network meta-analyses were evaluated (**Additional Reference 1: Appendix 6**). In addition, direct comparisons of the PSQI scores were evaluated. (**Additional Reference 1: Appendix 7.2**).

#### Quality of life

A total of 17 (41, 42, 53, 59, 77, 93, 96, 99–108) studies evaluated FACT-B in 1372 participants. Seven interventions were included in the NMA (**Figure 3C**): CG, AE, ME, CBT, YG, SE, and OE. CTE (MD, 11.39; 95%CI, 4.11-18.66), YG (MD, 11.28; 95%CI, 1.63-20.93), and AE (MD, 9.34; 95%CI, 0.26~18.42) demonstrated superior FACT-B improvement compared with CG. (**Figure 4C**). The comparison of the adjusted funnel plots did not provide evidence of significant publication bias, as confirmed by Egger’s test (*P* = 0.365) (**Additional document 1: Appendix 5.3**). Heterogeneity, inaccessibility, and inconsistencies in network meta-analyses were also evaluated (**Additional Reference 1: Appendix 6**). In addition, direct comparisons of FACT-B were assessed (**Additional Reference 1: Appendix 7.3**).

## Discussion

Breast cancer has the highest cancer incidence in women worldwide, and the survival rate of patients with breast cancer has been increasing. A range of side effects are often experienced by survivors of breast cancer, with CRF being a common side effect (109).

Several factors, including tumor stage, radiotherapy, chemotherapy, surgery, hormone therapy, radiation therapy dose, tumor burden, and the combination of these therapies, contribute to the development of CRF. Patients receiving cyclophosphamide, fluorouracil, adriamycin, or docetaxel experience more severe CRF than patients receiving paclitaxel alone (8, 110). An increase in the incidence of fatigue from 10% to 29% after treatment was observed in a cross-sectional study (111).

Furthermore, a patient’s CRF can be affected by factors such as family dysfunction, social support system, occupation, and hope level. Patients experiencing family dysfunction not only confront the physical pain induced by the disease and radiotherapy and chemotherapy but also endure the pressure arising from the family disorder, contributing to an increase in fatigue level (112). The onset of breast cancer and subsequent lifelong treatment impose medical burdens on patients, leading to emotional and role changes among some patient’s family members. This exacerbates the psychological burden and contributes to increased CRF (112). Sørensen HL et al. (113) discovered a close relationship between social support and physical and mental fatigue experienced by patients with breast cancer, with a stronger correlation observed for mental fatigue. Patients lacking social support face difficulties in receiving adequate support and assistance and struggle to effectively express sad emotions, leading to increased pressure on patients and a consequent worsening of fatigue. Yao Li et al. (114). found that farmers’ lower fatigue levels may be attributed to their low educational level, limited avenues for acquiring tumor-related knowledge, and higher expectations regarding disease prognosis. Furthermore, farmers may shoulder less social responsibility than patients with higher education, experiencing less social pressure, and exhibiting less noticeable fatigue. CRF has a tendency to persist and can result in dysfunction, reduced quality of life, and the emergence of negative emotions. Patients with breast cancer experience pain from CRF before or during treatment. Currently, a significant number of patients with breast cancer experience CRF, emphasizing the urgent need for its management.

Currently, numerous studies are focusing on improving CRF in breast cancer. This study conducted a literature search from 2001 to 2022, yielding 77 articles. Twelve various interventions (Aerobic exercise, Resistance training, Chinese traditional exercises, Other exercise, Multimodal exercise, Yoga, Stretching exercise, Music interventions, Cognitive behavioral therapy, Mindfulness training, Relaxation exercises, Control group) were analyzed to investigate their impact on patients with breast cancer and determine which intervention can effectively enhance CRF, alleviate depression, and improve quality of life in these patients.

The findings of this study indicate that CBT, CTE, AE, ME, MI, and YG were more effective than CG in improving CRF in patients with breast cancer. CBT can alter patients’ thinking, beliefs, and behaviors, correcting erroneous cognition and eliminating negative emotions through psychological treatment (115). Cognitive behavioral therapy can reduce CRF in patients with cancer through cognitive therapy, behavioral therapy, and psychological intervention. Clinical practice guidelines have recommended Cognitive behavioral therapy to reduce CRF in adults (116). The findings of this study are consistent with those of the traditional meta (117). In this study, we referred to Tai Chi, Qigong, and Baduanjin as Chinese traditional exercises. It was found that Chinese traditional exercises significantly improved CRF in patients with breast cancer compared to Control group. Tai Chi can regulate the nervous regions of the downstream stress response pathway by regulating the neuroendocrine system, including the autonomic nervous system and the hypothalamic-pituitary-adrenal axis. This regulation impacts the production of inflammatory factors (31, 118), leading to the downregulation of inflammatory factors, improvement in the inflammatory environment of patients’ tumors, and alleviation of CRF. Furthermore, Tai Chi is typically performed in groups, fostering a strong sense of community and social support among participating patients. They can share difficult everyday experiences and feel comfortable facing similar adversities. Additionally, social support can play a crucial role in buffering patients’ stress (119). Social support can enhance patients’ self-management ability, coping ability, and quality of life, including CRF (120). CTE regulates the respiratory system, enhances physiological function, promotes metabolism, and improves physical fitness, which can improve CRF in patients with breast cancer to a certain extent.

A relatively severe symptom burden caused by cancer diagnosis and treatment leads to patients being prone to sleep disorders (121). Sleep disorders affect more than 50% of patients with cancer, with some experiencing persistent and recurring sleep disorders within one year of completing treatment. Prolonged sleep disorders exacerbate patients’ pain, fatigue, psychological pain, and other symptoms, significantly affecting their quality of life and prognosis (122, 123).

This study discovered that Relaxation exercises, Music interventions, Aerobic exercise, and Multimodal exercise were more likely to improve the sleep quality of patients with breast cancer than Chinese traditional exercises, Other exercise, and Control group. Relaxation exercises regulates the function disturbed by tension stimulation, promotes muscle relaxation, reduces the arousal level of the cerebral cortex, and facilitates falling asleep through consciously repeated exercises of muscle tension and relaxation. Music interventions can affect the release of morphine peptides and other substances in the body and slow down negative emotions associated with sleep disorders, such as anxiety and depression, to improve sleep quality (117). Furthermore, Music interventions can regulate the body and mind of patients, inducing relaxation and guiding them into a relaxed and happy state, which contributes positively to stabilizing emotions and relieving pain. Relaxation exercises and Music interventions are also cost-effective and easy to practice. Using Relaxation exercises is recommended to improve sleep in patients with breast cancer and those experiencing sleep disorders. Moreover, this study observed that Chinese traditional exercises, Yoga, and Aerobic exercise were more effective than Control group in improving patients’ quality of life.

### Study strengths and limitations

This review has several advantages. First, mesh meta-analysis was used to directly and indirectly compare various interventions. Notably, more accurate interventions were included and meticulously classified into 12 various interventions, with each being defined. The effects of various intervention methods on CRF, PSQI, and quality of life were examined, along with the investigation of additional intervention measures. The findings of this study can be used as an optimal reference.

However, certain limitations exist in this study. First, the duration, intensity, and frequency of the interventions were not considered. Second, the implementation quality of the blind approach in the included literature is not high, and the outcome indicators are all subjective, lacking objective indicators. A description of the biological parameters should be added. Third, only Chinese–English literature was included, which may have resulted in heterogeneity. Fourth, all the studies were small sample studies; therefore, it is recommended to conduct future studies with large samples. Finally, in this study, the effects of tumor stage, patient treatment, patient’s psychological status, and patient’s family situation on CRF were not considered, which will have a particular impact on the results of this study.

## Conclusion

Evidence from systematic reviews and meta-analyses strongly recommends CBT for improving CRF in patients with breast cancer. RE and CTE are recommended to enhance the quality of sleep in patients with breast cancer. This study includes limited results, and it is recommended that future investigations include more studies to further validate the findings and select appropriate interventions based on the circumstances of patients with breast cancer.

## Data availability statement

The original contributions presented in the study are included in the article/**Supplementary Material**. Further inquiries can be directed to the corresponding author.

## Author contributions

YL: Writing – original draft, Writing – review & editing. LG: Data curation, Writing – original draft. RL: Data curation, Writing – original draft. JZ: Data curation, Software, Writing – original draft. ZZ: Data curation, Writing – original draft. SL: Writing – original draft, Data curation. XC: Supervision, Writing – original draft, Writing – review & editing. YC: Writing – review & editing, Supervision, Resources. TL: Writing – review & editing, Resources. JL: Writing – review & editing, Resources. ZW: Writing – review & editing, Supervision, Resources.
