# Supplementary material for: Various interventions for cancer-related fatigue in patients with breast cancer: a systematic review and network meta-analysis
Source: Front Oncol. 2024 Feb 9;14:1341927. doi: 10.3389/fonc.2024.1341927 (PMC10885696; doi:10.3389/fonc.2024.1341927)
Supplement: Supplementary file 1 [file DataSheet_1.pdf]

	Random sequence generation (selection bias)	Allocation concealment (selection bias)	Binding of participants and personnel (performance bias)	Binding of outcome assessment (detection bias)	Incomplete outcome data (attrition bias)	Selective reporting (reporting bias)	Other bias
Banasik 2011	+	+	?	?	+	+	?
Benben 2021	+	+	−	−	+	+	?
Boing 2017	−	−	−	−	+	+	?
Bolam 2019	+	+	−	−	+	+	?
Bower 2012	+	+	+	+	+	+	?
Bower 2015	+	+	+	+	+	+	?
Caoxin 2016	+	?	−	−	+	+	?
Changli 2016	+	+	−	−	+	+	?
Chaoul 2018	+	?	−	−	+	+	?
Chen 2013	+	+	?	?	+	+	?
Cohen 2021	+	+	?	?	+	+	?
Courneya 2007	+	+	+	+	+	+	?
Cramer 2015	+	+	+	+	+	+	?
Cuifeng 2017	+	+	−	−	+	+	?
Daley 2007	+	+	?	?	+	+	?
Danhauer 2009	+	+	−	−	+	+	?
Dieli 2018	+	+	−	−	+	+	?
Dongyang 2017	+	+	−	−	+	+	?
Duping 2019	+	+	−	−	+	+	?
Fong 2013	+	+	+	+	+	+	?
Gokal 2016	+	+	?	?	+	+	?
Guofei 2014	+	+	+	?	+	+	?
Hanqiong 2019	+	+	+	?	+	+	?
Haonan 2013	+	+	−	−	+	+	?
Huang 2016	+	+	−	−	+	+	?
Huiru 2022	−	?	−	−	+	+	?
Husebø 2014	+	+	+	?	+	+	?
Irwin 2017	+	+	+	?	+	+	?
Jiali 2018	+	+	−	−	+	+	?
Jinfang 2017	+	+	?	?	+	+	?
Jinmei 2021	+	+	−	−	+	+	?
Jong 2018	+	+	+	+	+	+	?
Junzhen 2019	+	+	−	−	+	+	?
Lengacher 2016	+	+	−	−	+	+	?
Liao 2022	+	+	−	−	+	+	?
Lijing 2019	+	+	−	−	+	+	?
Lina 2015	+	+	+	+	+	+	?
Liqun 2017	+	+	−	−	+	+	?
Liu 2022	+	+	+	+	+	+	?
Loh 2014	+	+	?	?	+	+	?
Lötzke 2016	+	?	−	−	+	+	?
Mijwel 2018	+	+	−	−	+	+	?
Moadel 2007	+	+	−	−	+	+	?
Mock 2001	+	+	?	?	+	+	?
Mock 2005	+	+	+	+	+	+	?
Naraphong 2014	+	+	+	+	+	+	?
Odynets 2019	+	+	+	+	+	+	?
Park 2020	+	+	−	−	+	+	?
Paulo 2018	+	+	−	−	+	+	?
Pinto 2005	+	+	?	?	+	+	?
Rahmani 2015	+	+	−	−	+	+	?
Reich 2014	+	+	−	−	+	+	?
Rogers 2015	+	+	+	+	+	+	?
Schad 2013	+	+	−	−	+	+	?
Schmidt 2015	+	+	+	+	+	+	?
Stan 2016	+	?	−	−	+	+	?
Steindorf 2014	+	+	+	+	+	+	?
Strunk 2018	+	+	?	?	+	+	?
Taohua 2020	+	+	−	−	+	+	?
Vadiraja 2009	+	+	?	?	+	+	?
Vadiraja 2017	+	?	−	−	+	+	?
Vardar 2015	+	?	−	−	+	+	?
Wang 2011	+	?	?	?	+	+	?
Wei 2022	+	+	?	+	+	+	?
Xierong 2022	−	−	−	−	+	+	?
Ximei 2021	−	−	−	−	+	+	?
Xiongping 2019	+	+	−	−	+	+	?
Xuying 2012	+	+	−	−	+	+	?
Yanbing 2022	+	+	−	−	+	+	?
YangLi 2022	+	+	−	−	+	+	?
Yangliu 2022	+	+	−	−	+	+	+
Yangmin 2020	+	?	?	?	+	+	?
Yingying 2017	+	+	+	+	+	+	?
Yongling 2021	+	?	−	−	+	+	?
Yuxin 2020	+	+	−	−	+	+	?
ZhangJiang2020	+	+	−	−	+	+	?
Zhiyuan 2019	+	+	−	−	+	+	?
