# Supplementary figures and images for: Various interventions for cancer-related fatigue in patients with breast cancer: a systematic review and network meta-analysis
Source: Front Oncol. 2024 Feb 9;14:1341927. doi: 10.3389/fonc.2024.1341927 (PMC10885696; doi:10.3389/fonc.2024.1341927)

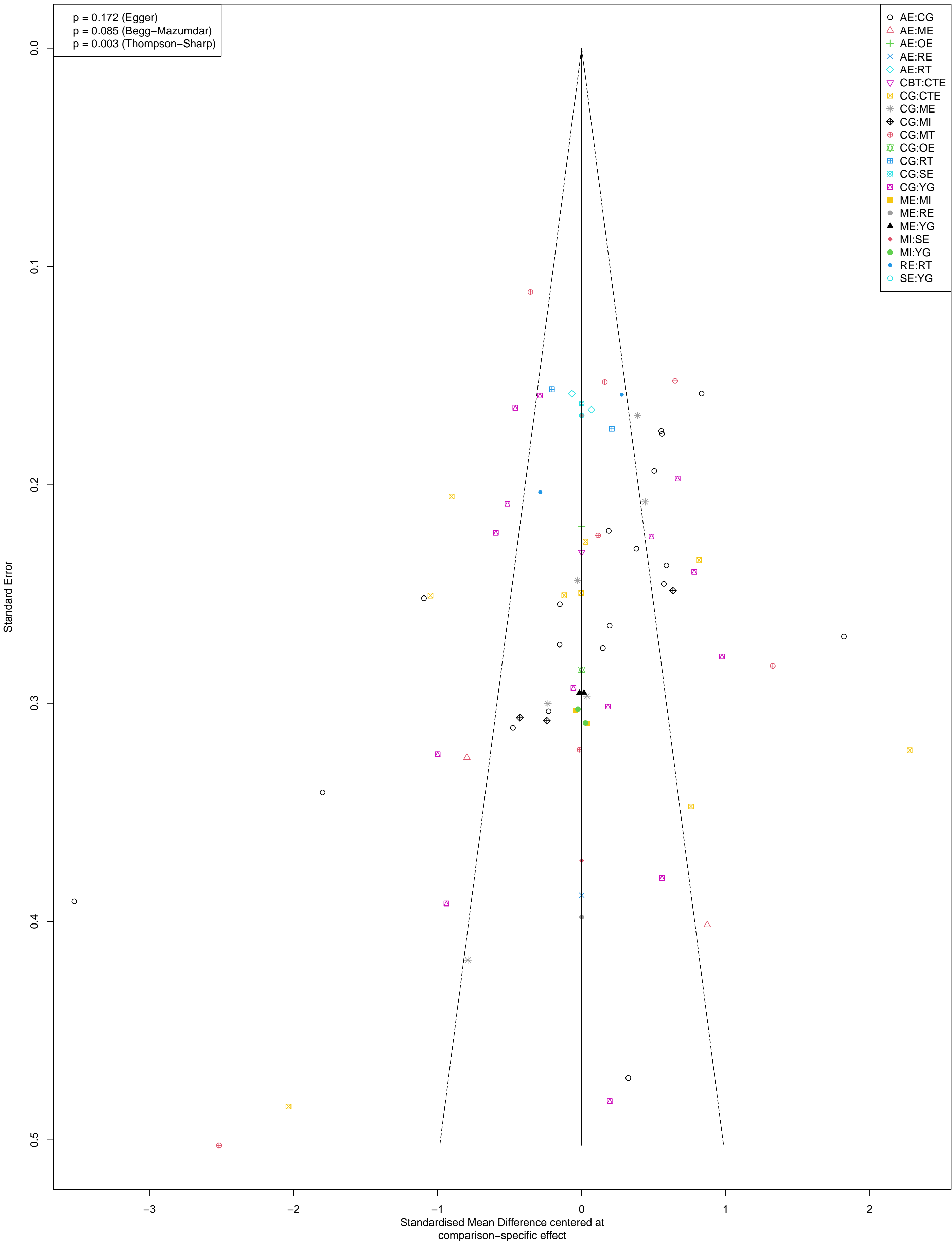

Supplement: Supplementary file 2 [file DataSheet_2.pdf]

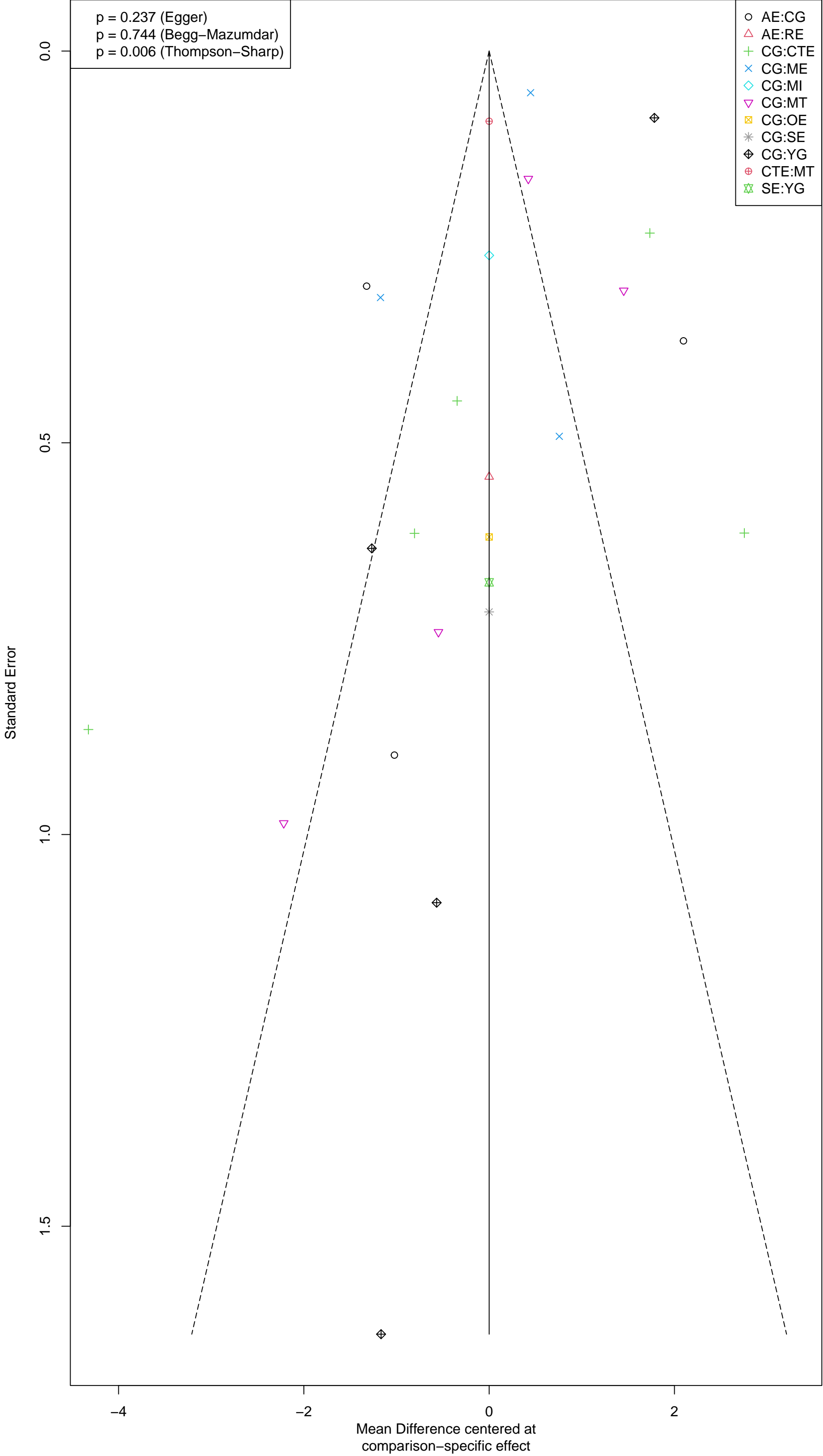

Supplement: Supplementary file 3 [file DataSheet_3.pdf]

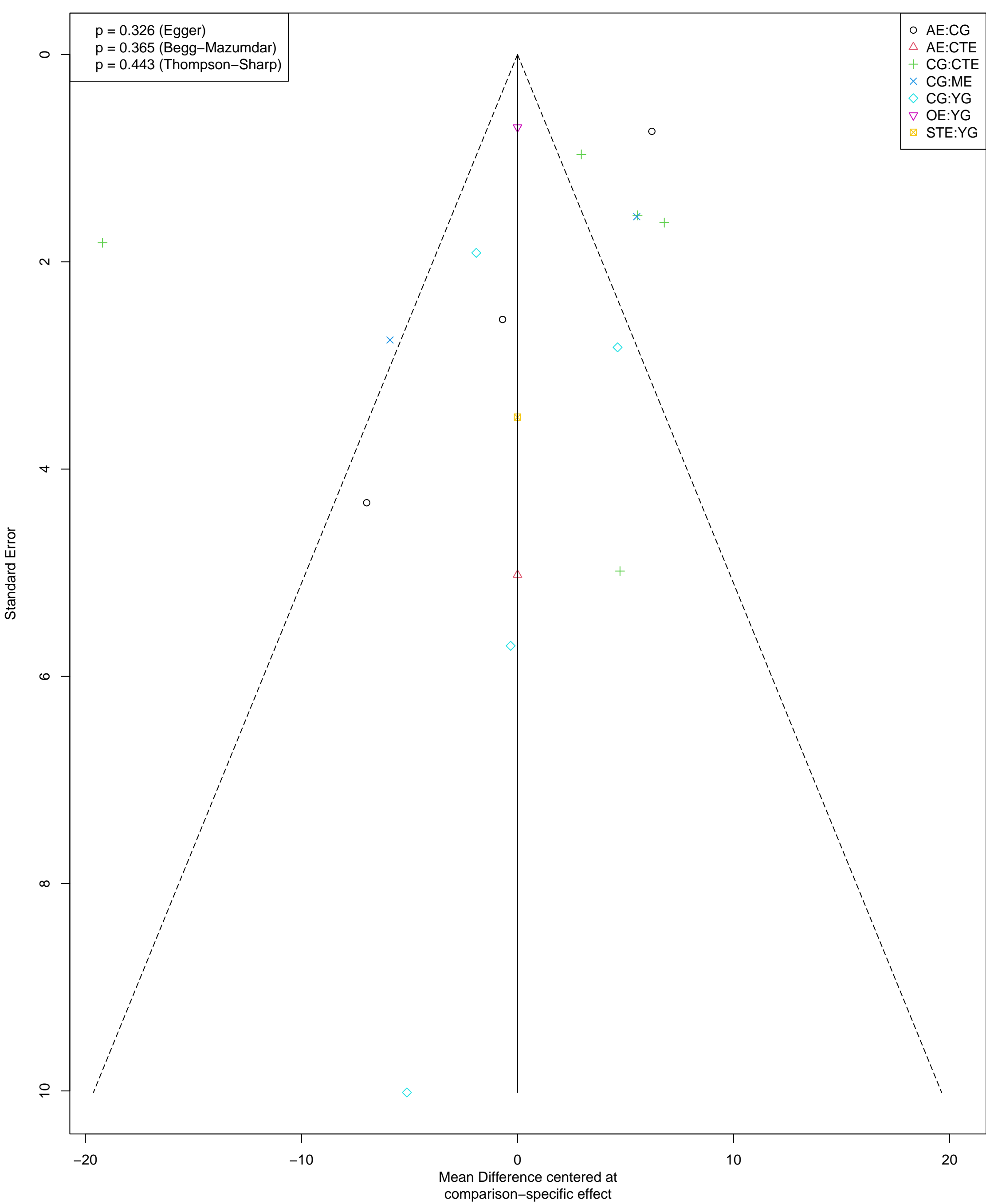

Supplement: Supplementary file 4 [file DataSheet_4.pdf]

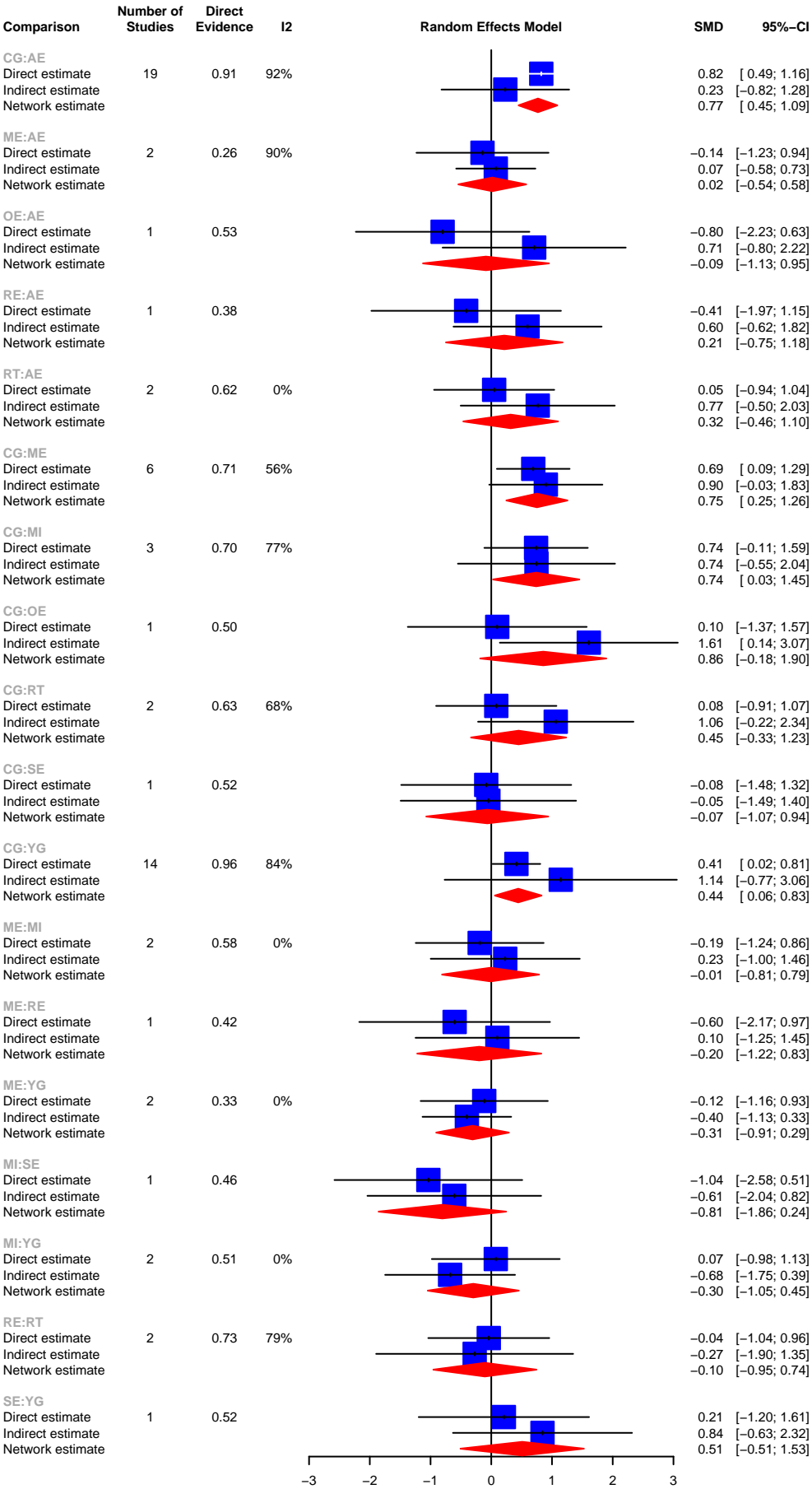

Supplement: Supplementary file 5 [file DataSheet_5.pdf]

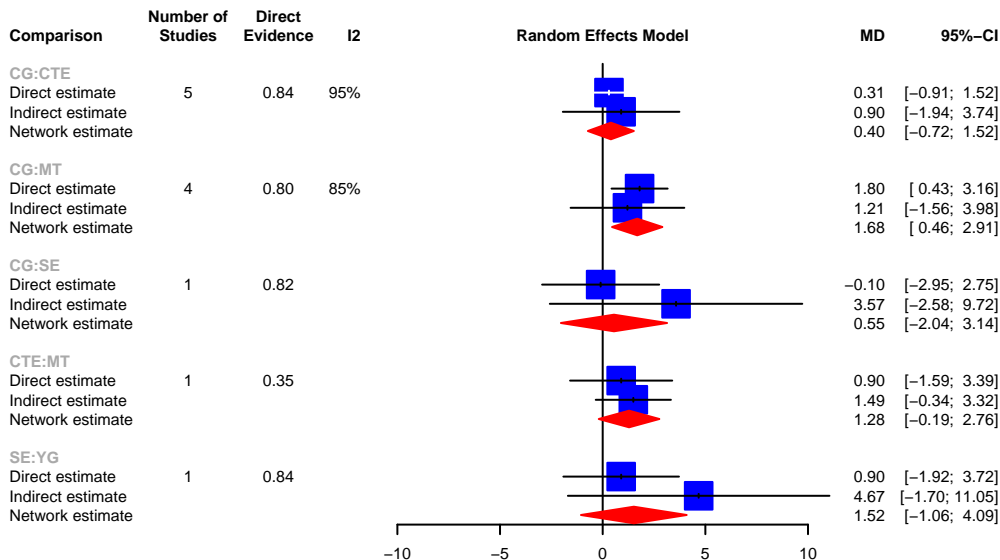

Supplement: Supplementary file 6 [file DataSheet_6.pdf]

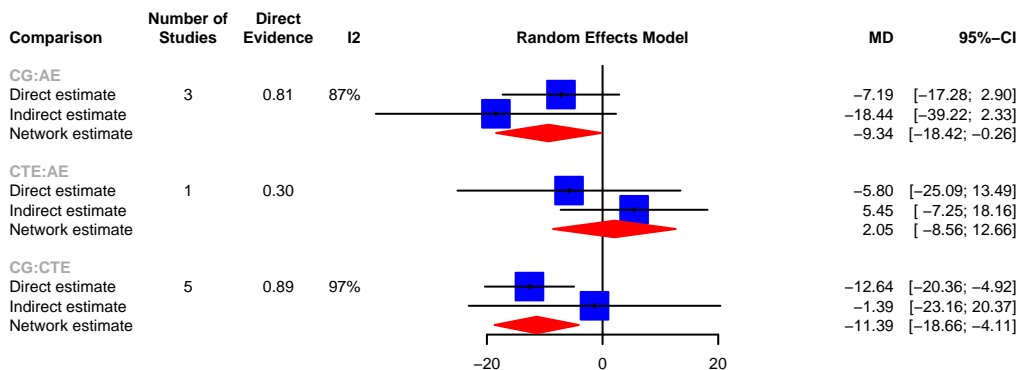

Supplement: Supplementary file 7 [file DataSheet_7.pdf]
