# Supplementary material for: Various interventions for cancer-related fatigue in patients with breast cancer: a systematic review and network meta-analysis
Source: Front Oncol. 2024 Feb 9;14:1341927. doi: 10.3389/fonc.2024.1341927 (PMC10885696; doi:10.3389/fonc.2024.1341927)
Supplement: Supplementary file 8 [file Table_1.docx]

**Pubmed:**

| Number | Search terms | Results |
| --- | --- | --- |
| **#1** | ((((((((((((((((((((((((((((((((((((Breast Neoplasm[MeSH Terms]) OR (Neoplasm, Breast[Title/Abstract])) OR (Breast Tumors[Title/Abstract])) OR (Breast Tumor[Title/Abstract])) OR (Tumor, Breast[Title/Abstract])) OR (Tumors, Breast[Title/Abstract])) OR (Neoplasms, Breast[Title/Abstract])) OR (Breast Cancer[Title/Abstract])) OR (Cancer, Breast[Title/Abstract])) OR (Mammary Cancer[Title/Abstract])) OR (Cancer, Mammary[Title/Abstract])) OR (Cancers, Mammary[Title/Abstract])) OR (Mammary Cancers[Title/Abstract])) OR (Malignant Neoplasm of Breast[Title/Abstract])) OR (Breast Malignant Neoplasm[Title/Abstract])) OR (Breast Malignant Neoplasms[Title/Abstract])) OR (Malignant Tumor of Breast[Title/Abstract])) OR (Breast Malignant Tumor[Title/Abstract])) OR (Breast Malignant Tumors[Title/Abstract])) OR (Cancer of Breast[Title/Abstract])) OR (Cancer of the Breast[Title/Abstract])) OR (Mammary Carcinoma, Human[Title/Abstract])) OR (Carcinoma, Human Mammary[Title/Abstract])) OR (Carcinomas, Human Mammary[Title/Abstract])) OR (Human Mammary Carcinomas[Title/Abstract])) OR (Mammary Carcinomas, Human[Title/Abstract])) OR (Human Mammary Carcinoma[Title/Abstract])) OR (Mammary Neoplasms, Human[Title/Abstract])) OR (Human Mammary Neoplasm[Title/Abstract])) OR (Human Mammary Neoplasms[Title/Abstract])) OR (Neoplasm, Human Mammary[Title/Abstract])) OR (Neoplasms, Human Mammary[Title/Abstract])) OR (Mammary Neoplasm, Human[Title/Abstract])) OR (Breast Carcinoma[Title/Abstract])) OR (Breast Carcinomas[Title/Abstract])) OR (Carcinoma, Breast[Title/Abstract])) OR (Carcinomas, Breast[Title/Abstract]) | 456,721 |
| **#2** | ((((((((((((((((((((resistance training[Title/Abstract]) OR (swimming[Title/Abstract])) OR (running[Title/Abstract])) OR (jogging[Title/Abstract])) OR (walking[Title/Abstract])) OR (yoga[Title/Abstract])) OR (sports[Title/Abstract])) OR (cycling[Title/Abstract])) OR (physical activity[Title/Abstract])) OR (aerobics[Title/Abstract])) OR (TaiJi[Title/Abstract])) OR (QiGong[Title/Abstract])) OR (Exercise[Title/Abstract])) OR (yoga[Title/Abstract])) OR (Activity[Title/Abstract])) OR (Physical[Title/Abstract])) OR (Ba Duan Jin[Title/Abstract])) OR (mindfulness[Title/Abstract] OR (music[Title/Abstract] )) | 4,271,464 |
| **#3** | #1 AND #2 | 65,708 |
| **#4** | ((fatigue [Title/Abstract]) OR (Lassitude [Title/Abstract])) OR (cancer-related fatigue OR [Title/Abstract]) | 127,151 |
| **#5** | #3 AND #4 | 1962 |
